# Predictors of outcome after a time-limited psychosocial intervention for adolescent depression

**DOI:** 10.3389/fpsyg.2022.955261

**Published:** 2022-11-02

**Authors:** Pauliina Parhiala, Mauri Marttunen, Vera Gergov, Minna Torppa, Klaus Ranta

**Affiliations:** ^1^Department of Adolescent Psychiatry, Helsinki University Hospital and University of Helsinki, Helsinki, Finland; ^2^Faculty of Social Sciences, University of Tampere, Tampere, Finland; ^3^Department of Public Health Solutions, Finnish Institute for Health and Welfare in Finland (THL), Helsinki, Finland; ^4^Faculty of Medicine, University of Helsinki, Helsinki, Finland; ^5^Department of Teacher Education, University of Jyväskylä, Jyväskylä, Finland

**Keywords:** adolescent, depression, brief intervention, school mental health services, symptom improvement, predictors

## Abstract

Research on the predictors of outcome for early, community-based, and time-limited interventions targeted for clinical depression in adolescents is still scarce. We examined the role of demographic, psychosocial, and clinical variables as predictors of outcome in a trial conducted in Finnish school health and welfare services to identify factors associating to symptom reduction and remission after a brief depression treatment. A total of 55 12–16-year-olds with mild to moderate depression received six sessions of either interpersonal counseling for adolescents (IPC-A) or brief psychosocial support (BPS). Both interventions resulted in clinical improvement at end of treatment and 3- and 6-month follow-ups. Main outcome measures were self-rated BDI-21 and clinician-rated Adolescent Depression Rating Scale (ADRSc). Latent change score (LCS) models were used to identify predictors of change in depressive symptom scores and clinical remission at end of treatment and 3- and 6-month follow-ups over the combined brief intervention group. Symptom improvement was predicted by younger age and having a close relationship with parents. Both symptom improvement and clinical remission were predicted by male gender, not having comorbid anxiety disorder, and not having sleep difficulties. Our results add to knowledge on factors associating with good treatment outcome after a brief community intervention for adolescent depression. Brief depression interventions may be useful and feasible especially for treatment of mild and moderate depression among younger adolescents and boys, on the other hand clinicians may need to cautiously examine sleep problems and anxiety comorbidity as markers of the need for longer treatment.

## Introduction

Depression is highly prevalent and impairing disorder in adolescents across the globe, yet it often goes unrecognized and undertreated (Haarasilta et al., 2003; Merikangas et al., 2010; Jörg et al., 2016; Zuckerbrot et al., 2018). Left untreated, depression adds to the risk of functional impairment and compromised physical and mental health in adulthood (Zisook et al., 2007; Thapar et al., 2012).

Research suggests that intervening early to symptoms of depression is associated with higher effectiveness (Horowitz and Garber, 2006; Bertha and Balázs, 2013) and cost-effectiveness (Chisholm et al., 2016) of interventions. Access to mental healthcare, implementation of mental health programs and insufficient mental health policies challenges the provision of early interventions for clinical depression in adolescents (Rocha et al., 2015). Due to limited knowledge on outcomes and predictors of outcomes of early interventions research on them seems warranted (Davey and McGorry, 2019).

Structured psychotherapies are considered first-line interventions for adolescent depression (NICE, 2019). Short term cognitive behavioral therapy (CBT) and interpersonal psychotherapy for adolescents (IPT-A) have gained most research support of efficacy for adolescent depression (Birmaher et al., 2007; Weisz et al., 2013, 2017; Zhou et al., 2015; Pu et al., 2017). Dissemination of such treatments and use in primary care and community services is often called for, because structural and organizational factors in public healthcare systems often prevent delivery of longer treatments, which often require extensive mental health training for the professionals (Flaherty et al., 2021).

Preliminary evidence of feasibility and positive pre-to-post treatment effect for depression in adolescents have been found for short forms of interpersonal therapy, i.e., Interpersonal Counseling for Adolescents, IPC-A (Wilkinson et al., 2018; Parhiala et al., 2020), and Brief Interpersonal Therapy for Adolescents, BIPT-A (Mufson et al., 2015) in community settings. National and professional guidelines suggest using supportive counseling either in individual or group format in primary care settings and schools for treatment of mild depression in adolescents (Lewandowski et al., 2013; Cheung et al., 2018; NICE, 2019).

Even when adequately administered, interventions for depression are effective for only 50–70% of treated adolescents, and about 30–40% achieve remission (e.g., Emslie et al., 2003; March et al., 2004; Thapar et al., 2012; Weisz et al., 2017). Identification of individual and psychosocial factors associated with good treatment outcomes for interventions of varying length is important to policymakers and clinical directors for allocating financial resources and for constructing stepped care treatment models (Berger et al., 2021).

Effective use of available resources requires knowledge on for whom brief interventions are likely to be sufficient, and who need longer treatments. In clinical intervention research, pre-treatment factors associated with treatment outcome, independent of the treatment condition given, are called predictor variables (Barber, 2009). Predictors can be grouped under broader categories in several ways (e.g., Vousoura et al., 2021) with one categorization presented below.

### Demographic variables

Clinical research shows that age, gender, and family socioeconomic status might influence psychotherapy treatment outcomes for adolescent depression. In a recent meta-analysis of psychotherapies for depression, Cuijpers et al. (2020) found that children under the age 13 showed poorer treatment outcomes than adolescents between ages 13 and 18 regardless of the type of therapy. Contrasting this, an earlier meta-analysis (Weisz et al., 2006) found age was not significantly associated with outcome of psychotherapy of child and adolescent depression.

Results from trials of CBT, supportive therapy (Clarke et al., 1995; Brent et al., 1998; Jayson et al., 1998; Curry et al., 2006), and family therapy (Brent et al., 1998; Diamond et al., 2019) among depressed adolescents suggest that younger adolescents may have stronger response to psychotherapy than older adolescents. In contrast, Mufson et al. (2004) found symptomatic improvement greater in older, compared with younger adolescents after IPT-A.

Most meta-analyses of adolescent depression trials have not found gender to have an effect on treatment outcome (Clarke et al., 1992; Curry et al., 2006; Weisz et al., 2006; Courtney et al., 2022). However, in an early meta-analysis, Weisz et al. (1995) reported adolescent girls to benefit more from psychotherapy than boys, while among prepubertal children the gender effect was not found. The scoping review by Courtney et al. (2022) suggests that socioeconomic status is not a powerful predictor of outcome in adolescent depression.

### Psychosocial variables

As parental behaviors and family problems commonly associate with depression in the young (Feeny et al., 2009), family factors can be expected to have influence on treatment outcomes. Indeed, living in a single-parent household was found to be a predictor of poorer depression treatment outcome in a study by Brent et al. (1998). Furthermore, maternal report of parent–adolescent conflict (Feeny et al., 2009), high family conflict (Asarnow et al., 2009), impairment in social functioning (Jayson et al., 1998), and high overall social dysfunction within the family (Gunlicks-Stoessel et al., 2010), have been found to predict poorer outcomes for adolescent depression treatment, irrespective of the type of treatment received.

### Clinical variables

Studies examining the effects of clinical variables as predictors of treatment outcomes with depressed adolescents have found baseline severity of depression to predict poorer treatment outcomes across different types of interventions (Brent et al., 1998; Jayson et al., 1998; Mufson et al., 2004; Asarnow et al., 2009; Wilkinson et al., 2009). According to a review by Nilsen et al. (2013), majority of reviewed studies found high severity of depression at baseline to predict poorer treatment response.

In the review by Emslie et al. (2011) depressed adolescents having any comorbid psychiatric disorder had poorer outcomes in the included depression treatment studies. Of comorbid disorders, anxiety disorders were the most common predictors of poor treatment outcome. It have been found to predict poorer treatment outcome irrespective of severity of depression in CBT and IPT-A trials (Curry et al., 2006; Young et al., 2006; Wilkinson et al., 2009) and in a family therapy trial (Diamond et al., 2019). In two studies, comorbid anxiety disorders did not predict treatment outcome for adolescent depression (Jayson et al., 1998; Rohde et al., 2001). In the Rohde et al. (2001) study, higher depression severity in those with comorbid disorders ruled out the effect of anxiety disorders.

Sleep difficulties commonly precede depressive episodes in adolescents and predict onset of depression in longitudinal studies (Lovato and Gradisar, 2014). According to Kennard et al. (2006) sleep disturbance was one of the most common residual symptoms in adolescents who were fully or partly remitted from MDD after receiving CBT. Yet, sleep difficulties have relatively rarely been studied as a predictor of adolescent depression treatment outcome.

In adolescents treated with fluoxetine, Emslie et al. (2012) found that pretreatment insomnia had a significant negative impact on treatment response and remission. In a continuation study of youth with MDD who had responded to acute treatment with fluoxetine (Kennard et al., 2018), residual insomnia after acute treatment predicted almost sevenfold risk of relapse. In a trial of IPT-A and routine treatment McGlinchey et al. (2017) found sleep disturbance to predict worse outcome in adolescent depression irrespective of treatment type.

To sum, research on predictors of outcome from trials of psychosocial treatments for adolescent depression show heterogeneous results. Most previous studies have been conducted in university clinics or specialized mental health clinics, generally treating adolescents with severe disorders. It is not clear whether factors associated with positive treatment outcomes are the same when adolescent depression is treated in community, or in school health and welfare services with time-limited interventions, by professionals not having extensive background training in mental health.

To add to the literature, we studied predictors of outcome in a clinical trial comparing interpersonal counseling for adolescents (IPC-A) with brief psychosocial support (BPS), delivered in school health and welfare services (Parhiala et al., 2020). In the trial both treatments were effective with no statistically significant differences between the treatments on outcome. Both treatments were brief (i.e., six sessions) and feasible to implement in community services. Thus, IPC-A and BPS treatment arms were combined in the predictor analyses, and treatment modality (IPC-A or BPS) was used as a covariate in the analyses.

The aim of this study was to examine selected baseline demographic, psychosocial and clinical variables, identified from previous research, as possible predictors of outcome. Based on extant literature we expected that younger age would predict better treatment outcome and gender would have no effect on the outcome. We further expected that positive relationship between the adolescent and parents would be associated with a positive outcome. Third, we hypothesized that adolescents with milder depression, no comorbid anxiety disorder and no sleep difficulties at baseline would respond better to a brief intervention.

## Materials and methods

### Procedure and recruitment

The trial from which our data is drawn compared two brief interventions for adolescent depression, Interpersonal counseling for adolescents (IPC-A) and brief psychosocial support (BPS) in Finnish school health and welfare services (Parhiala et al., 2020). All Finnish secondary schools provide student health and welfare services, their staff consisting of school psychologists, social workers, and nurses. In these services, adolescents are provided psychosocial support on as need basis. Those with need of prolonged support or identified mental health disorders are typically referred to specialized mental health services. A cluster randomization design was used. The participating schools were randomized to provide six sessions of either IPC-A or BPS. Outcome measures were given at baseline (session 1), mid-treatment (session 4), end of treatment (session 6), and follow-up meetings (3- and 6-month follow-up).

The recruitment process followed routine practice for adolescents to obtain support from school services. Participants were screened for eligibility to the study using the Finnish modification of the 13-item Beck Depression Inventory, R-BDI (Beck and Beck, 1972; Raitasalo, 2007). Those who screened positive (R-BDI sum score > 5) and consented were referred to diagnostic interview. For a more detailed description (e.g., flow chart, study design, referral process), see Parhiala et al. (2020).

### Participants

Fifty-five 12–16-year-old students were recruited from the student health and welfare services of the public lower secondary schools of a city of approximately 250,000 inhabitants in Southern Finland. Of the participants, 43 were girls and 12 were boys. Their mean age (*SD*) was 14.53 (0.78) years.

### Measures

#### Symptom measures

The Beck Depression Inventory (BDI-21; Beck et al., 1961) was used as a measure of self-reported depressive symptoms. It has demonstrated good psychometric properties in previous studies in adolescents (Brooks and Kutcher, 2001; Myers and Winters, 2002). In the present study, internal consistency of BDI-21, measured by Cronbach’s alpha (α) was 0.89. The Adolescent Depression Rating Scale (ADRSc; Revah-Levy et al., 2007) was used as the measure of clinician-rated depression symptoms. The ADRSc was administered by the school professionals delivering the interventions. According to a previous study, ADRSc has good convergent, discriminant, and factorial validity to assess depression in adolescents (Revah-Levy et al., 2007). In the present study, internal consistency of ADRSc was 0.80. Both measures were administered at baseline (session 1), in mid-treatment (session 4), and at the end of treatment (session 6), and in both follow-up meetings (3- and 6-months after the end of treatment).

#### Diagnostic interview

The Schedule for Affective Disorders and Schizophrenia for School-Age Children (K-SADS-PL; Kaufman et al., 1997), version for DSM-5 (K-SADS-5), was administered by a clinical psychologist to assess present and lifetime mental health disorders according to Diagnostic and Statistical Manual of Mental Disorders (DSM-5). Diagnostic evaluation was administered during baseline and again at 3- and 6- month follow-ups. The psychometric properties of the instrument (DSM-IV) have been good (Kaufman et al., 1997). All adolescents receiving a diagnosis of mild or moderate MDD, dysthymia, or depressive disorder not otherwise specified, were offered to be included in the study. Exclusion criteria were severe psychiatric disorder, ongoing psychiatric treatment, and an acute need for child protection services. Four adolescents met the exclusion criteria (one severe major depression, one acute need for child protection services, one with primary and severe anxiety disorder, one with a psychotic disorder) and were referred to a service they needed.

### Treatments

Interpersonal counseling is a brief individual treatment focusing on current symptoms of depression in an interpersonal context (Weissman and Verdeli, 2013). In this study, IPC was delivered in six 45-min sessions over a 6–12-week period. The treatment was administered according to the procedures specified in the IPC treatment manual (Weissman and Verdeli, 2013), and its adaptation for adolescents (IPC-A; Wilkinson and Cestaro, 2015). A 3-day IPC-A training was given to all school health and welfare professionals at schools randomized to give IPC-A prior to onset of study. In addition, professionals got ongoing clinical method-specific supervision every second week for the duration of the trial.

Brief psychosocial support was based on the methods and techniques used by the school health and welfare professionals in their routine work. At BPS sites, the professionals delivered BPS without specific methodological training. However, they were instructed to target the intervention to symptoms of depression, and to assess depressive symptoms repeatedly. To ensure comparability across treatments, BPS was delivered with the same frequency and session duration as IPC-A.

Before the trial, all participating school health and welfare professionals were given 1-day training course in the identification and assessment of depression and the use of assessment measures included. Professionals in both treatment arms were instructed to assess and monitor symptoms of depression in their adolescent patients systematically and repeatedly.

### Predictors of treatment outcome

Due to the relatively small sample size, we limited potential predictors to the most relevant based on previous literature. We ended up to seven putative predictor variables in three categories: demographic variables, psychosocial variables, and clinical variables. These baseline predictor variables are described in Table 1.

**TABLE 1:** Predictor variables: Descriptive characteristics at baseline, post-treatment, and follow-ups.

	Variable	Baseline *N* = 55	Post-treatment *N* = 51	3-month follow-up *N* = 49	6-month follow-up *N* = 49
Demographic variables	Age: mean (*SD*)	14.53 (0.78)	14.52 (0.73)	14.48 (0.71)	14.48 (0.71)
	Female gender, *n* (%)	43 (78.2%)	40 (78.4%)	39 (79.6%)	39 (79.6%)
Psychosocial variables	Family constellation (living with both biological parents), *n* (%)	28 (50.9%)	27 (52.9%)	26 (53.1%)	26 (53.1%)
	Close relationship with parents, *n* (%)	41 (74.5%)	40 (78.4%)	39 (79.6%)	39 (79.6%)
Clinical variables	Comorbid anxiety disorder, *n* (%)	16 (29.1%)	15 (29.4%)	15 (30.6%)	15 (30.6%)
	Sleep difficulties, *n* (%)	10 (18.2%)	10 (19.6%)	9 (18.4%)	9 (18.4%)
	Self-reported depressive symptoms, BDI-21, mean (*SD*)	17.47 (9.12)	10.54^a^ (9.00)	11.21^a^ (11.29)	7.56^a^ (8.40)
	Clinician-reported depressive symptoms, ADRSc, mean (*SD*)	16.31^a^ (7.78)	9.47 (8.16)	10.80 (8.58)	7.57 (7.05)

BDI-21, Beck Depression Inventory; ADRSc, Adolescent Depression Rating Scale, clinician version. ^a^Data missing in one case.

Age and gender were used as demographic variables. Of psychosocial variables, we included family constellation (living with one or both biological parents) and the closeness of adolescent’s relationship with parents. Adolescents were asked about their relationship with parents by a question “how do you perceive your relationship with your mother and father at the moment.” When the adolescent reported both relationships were close (e.g., warm, easy to talk with parent), or close enough (e.g., no problems but not talking about everything with parent) this variable was coded as “close.” If the adolescent reported that the relationship was close with one parent, but not with the other (e.g., frequent conflicts, not feeling good about sharing feelings with parent), or if the adolescent felt the relationship was not close with either parent, the variable was coded as “not close.”

Of clinical variables, we included baseline severity of depressive symptoms, comorbid anxiety disorders, and sleep difficulties. Severity of baseline depressive symptoms was defined using both the continuous BDI-21 score and the ADRSc score. The BDI-21 scores were categorized into three groups according to symptom severity: (1) no/minimal depressive symptoms (0–9 points), (2) mild depressive symptoms (10–18 points), (3) moderate depressive symptoms (19 points or more) (Beck et al., 1988). The ADRSc baseline scores were classified into three severity levels: 1. no clinical depression (0–14 points), clinical depression (15–19 points), severe depression (20 points or more), as defined by Revah-Levy et al. (2007).

The presence of comorbid anxiety disorders and sleep difficulties were drawn from the K-SADS-5 interview. The presence of sleep difficulties was defined as either initial, middle, or terminal phase difficulty in getting to sleep or staying asleep, or hypersomnia. The participant was classified as suffering from sleep difficulties if he/she reported symptoms nearly every night (i.e., five to seven nights per week), including any type of sleep symptoms.

### Statistical analysis

**FIGURE 1:**
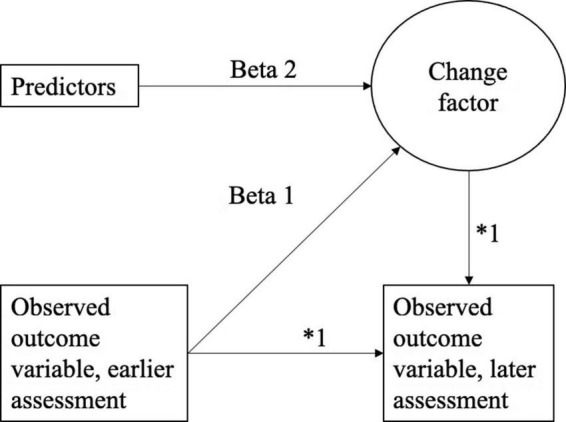
Latent change score (LCS) model specification.

The relationship between each predictor variable and observed change in depressive symptoms were tested with Latent Change Score (LCS) models. In the LCS models (Figure 1) two assessments of the observed outcome variables (BDI-21 and ADRSc), were included in each model. Therefore there are three models for BDI-21 and ADRSc. In the LCS models 1, the change between baseline and post-treatment (post) was modeled. In the LCS models 2, the change between post-treatment and 3-month follow-up (3-mo) was modeled, and in the LCS models 3, the change between 3- and 6-month follow-up (6-mo) was modeled. In the LCS models, the change between the two timepoints is modeled as latent variable. Predictors of the latent change were baseline variables including all tested variables in a certain model (intervention group, age, gender, family constellation, relationship with parents, anxiety disorders, sleep difficulties). The autoregressive parameters and factor loadings were fixed to one (marked as * in Figure 1). The standardized beta values for the models are reported in Table 2 and in Supplementary Table 1.

**TABLE 2:** The standardized estimates of the final latent change score (LCS) model for Beck Depression Inventory (BDI)-21 and Adolescent Depression Rating Scale (ADRSc).

	Post BDI-21 β (*s.e*.) R2	3-mo BDI-21 β (*s.e*.) R2	Post ADRSc β (*s.e*.) R2	3-mo ADRSc β (*s.e*.) R2
Depressive symptoms	–0.46 (0.08)*** 0.21	–0.66 (0.08)*** 0.44	–0.57 (0.09)*** 0.32	–0.76 (0.09)*** 0.58
Intervention group	0.07 (0.11) 0.00	0.08 (0.10) 0.01	0.16 (0.10) 0.03	0.00 (0.10) 0
Age	0.29 (0.13)* 0.08			
Gender	0.36 (0.10)** 0.13		0.25 (0.09)** 0.06	
Close relationship with parents		–0.22 (0.10)** 0.05		–0.17 (0.10) 0.03
Comorbid anxiety disorder			–0.24 (12)* 0.06	
Sleep difficulties			0.39 (0.11)*** 0.15	
R2	0.37	0.50	0.49	0.60
Model fit	χ^2^(3) = 4.27, *p* > 0.05, *RMSEA* = 0.09, *CFI* = 0.96, *SRMR* = 0.10	χ^2^(2) = 1.80, *p* > 0.05, *RMSEA* = 0.00, *CCFI* = 1.00, *SRMR* = 0.09	χ^2^(4) = 8.68, *p* > 0.05, *RMSEA* = 0.15, *CFI* = 0.90, *SRMR* = 0.16	χ^2^(2) = 0.36, *p* > 0.05, *RMSEA* = 0.00, *CFI* = 1.00, *SRMR* = 0.04

Post, change in depression score from baseline to post-treatment; 6-mo, change in depression score from 3- to 6 month follow-up; BDI-21, Beck Depression Inventory; ADRSc, Adolescent Depression Rating Scale clinician version; β, standardized estimate for regression; s.e., standard error; R2, amount of explained variance. *p < 0.05, **p < 0.01, ***p < 0.001.

The LCS models were run for both BDI-21 and ADRSc scores to explore the person-to-person variability in the change of depressive symptoms during the intervention and follow-up points. In the initial LCS model, all predictor variables were included in the model: the demographic variables (age, gender), the psychosocial variables (family constellation, relationship with parents), and the clinical variables (baseline severity of depression symptoms, comorbid anxiety disorder, sleep difficulties). Intervention type (IPC-A or BPS) was used as a covariant in the analyses. In the second, final LCS model, only the statistically significant predictor variables were included. Due to sample size, the LCS models were run separately for the three separate time periods: Post (change in depression score from first treatment session to last session), 3-mo (change in depression score from last session to 3-month follow-up), and 6-mo (change in depression score from 3- to 6-month follow-up).

One-way analyses of variance (ANOVA) were conducted to compare gain-scores (i.e., decrease or increase of the depression score during Change period) in groups categorized according to baseline BDI-21 and ADRSc throughout the treatment and follow-ups. Direction of change was analyzed using Dunnett’s correction separately for BDI-21 and ADRSc.

Last, we analyzed whether the chosen baseline variables predicted clinical remission according to BDI-21 and ADRSc at post-treatment, at 3- and at 6-month follow-up, using Chi-square tests for nominal variables and *t*-tests for continuous data (i.e., age). We also checked whether depression symptom scores were different between the two comparison groups within each predictor variable already at baseline, using *t*-tests. In these analyses, nominal variables were compared, and continuous variable (i.e., age) was divided into two groups using the mean value. Clinical remission was defined as the absence of clinically significant depressive symptoms score < 10 in BDI (Beck et al., 1988) and score < 15 in ADRSc (Revah-Levy et al., 2007). Missing data were imputed by carrying the last observation forward until the sixth session if the adolescent had at least one completed BDI-21 or ADRSc after baseline. Data analyses were carried out using SPSS (version 22.0) and Mplus (version 8; Muthén and Muthén, 1998-2017) programs.

## Results

Descriptive data on baseline predictor variables are presented in Table 1. Examination of depressive symptoms at baseline showed that participants suffered from moderate depressive symptoms according to BDI-21 scores (*M* = 17.47, *SD* = 9.12). According to ADRSc sores their symptoms were in the clinical depression range (*M* = 16.10, *SD* = 7.78). As shown by standard deviations, variation in scores was high. Depression scores decreased during the intervention, but variation between participants remained large through the duration of intervention and follow-ups (see Table 1).

Four participants dropped out from the treatment after the third session, and one participant did not answer the BDI-21 questionnaire after the baseline. Therefore, the number of participants included in the BDI-21 analyses was 50 at the end of treatment, for ADRSc it was 51. Two participants dropped out after the fifth session and one participant did not answer the BDI-21 questionnaire at the 3-month follow-up. Thus, the number of adolescents included in the 3-mo analyses of BDI-21 is 48 and 49 in the ADRSc analyses.

### Predicting change in depressive symptoms during the intervention

The initial LCS models including all predictor variables is presented in Supplementary Table 1. Separate models were run for BDI-21 and ADRSc scores. Table 2 presents the final LCS models including only the statistically significant predictors and the intervention type as a covariant, separately for BDI-21 and ADRSc scores. The amount of explained variance in the change factor is reported in the above tables. Note that the amount of unique variance explained by each predictor equals to the squared standardized path estimates (betas).

#### Change in self-reported depressive symptoms (Beck Depression Inventory-21)

The results from LCS models (Table 2 and Supplementary Table 1) showed that the previous BDI-21 score significantly predicted the subsequent BDI-21 score at each of the three time periods examined (i.e., post, 3-mo, 6-mo).

**FIGURE 2:**
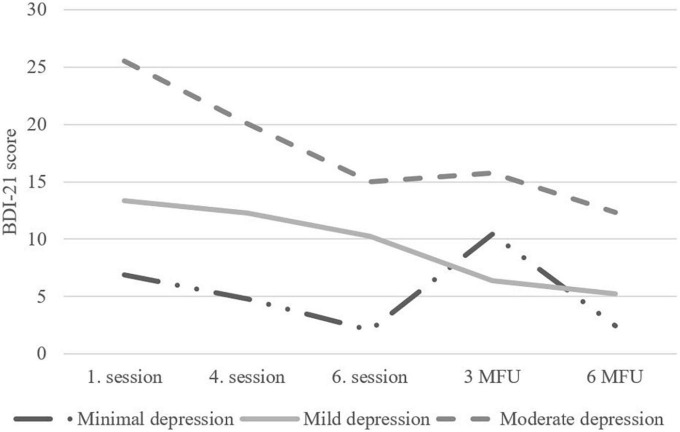
Change in clinical severity groups according to Beck Depression Inventory (BDI)-21-scores from baseline to 6-month follow-up.

A larger decrease in BDI-21 was found for adolescents with higher BDI-21 baseline scores (Figure 2). In the post model, a larger decrease in BDI total score was predicted by younger age and male gender. The initial model for 3-mo resulted in no significant predictors of BDI change. The model for 6-mo indicated that a larger decrease in BDI-21 total score between the 3- and 6-month follow-up assessments was predicted by having close relationships with parents. The initial model for post and 6-mo had insufficient model fit, as there were too many variables in comparison to the small number of adolescents, but the final models for post and 6-mo fitted the data well (Table 2 and Supplementary Table 1). The initial model for 3-mo did not fit the data well, as none of the examined predictors explained the change. Thus, final model for 3-mo was not run.

#### Change in clinician-rated depressive symptoms (Adolescent Depression Rating Scale)

**FIGURE 3:**
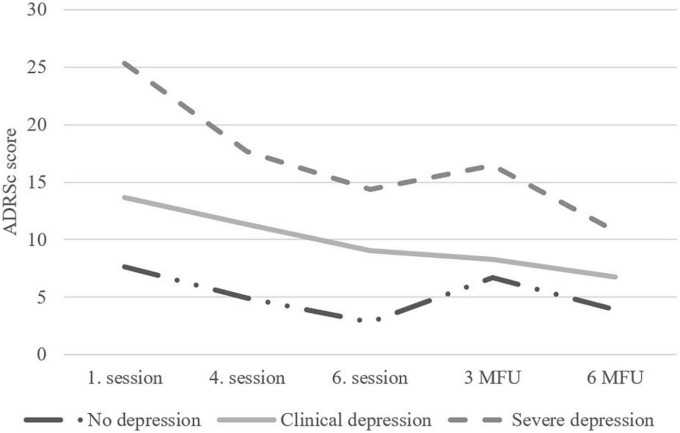
Change in clinical severity groups according to Adolescent Depression Rating Scale (ADRSc)-scores from baseline to 6-month follow-up.

The results from the LCS models (Table 2 and Supplementary Table 1) showed that the previous ADRSc score significantly predicted the subsequent ADRSc score in post and 6-mo models. A larger decrease in ADRSc was found for adolescents with higher ADRSc baseline scores (Figure 3). In the post model, a larger decrease in ADRSc total score was predicted by male gender and not having sleep difficulties. Not having comorbid anxiety disorder was almost significant (*p* = 0.059) in the initial LCS model including all predictor variables. It was therefore also included in the final model and predicted change of ADRSc sum score in the final model. In the initial LCS model for 6-mo close relationship with parents was almost significant (*p* = 0.054) and was included in the final model. In the final model it was not, however, a significant predictor. The model for 3-mo resulted in no significant predictors of ADRSc change. The initial models for post and 6-mo had insufficient model fit, as there were too many variables in comparison to the small number of adolescents, but the final models for post and 6-mo fitted the data well (Table 2 and Supplementary Table 1). The initial model for 3-mo did not fit the data well, as none of the predictors explained the change. Thus, the final model for 3-mo was not run.

#### Change according to clinical severity

The LCS models suggested that baseline depression scores predicted changes during the intervention and follow-up. To examine the effect more closely, we analyzed how the baseline scores categorized according to clinical severity of symptoms BDI-21: no/minimal symptoms, mild symptoms, moderate symptoms (Beck et al., 1988) ADRSc: no clinical depression, clinical depression, severe depression (Revah-Levy et al., 2007) affected the outcome at the end of treatment and follow-up. As can be seen from Figures 2, 3, the depression scores of participants in the moderate symptoms (BDI-21) or severe depression (ADRSc) groups decreased rapidly. However, these adolescents still ended up having higher scores during the follow-up in comparison to those with lower scores at baseline.

We compared the three baseline depression symptom and depression severity groups (see Section “Predictors of treatment outcome”) according to baseline BDI-21 and ADRSc using gain-scores in three analyses: (1) between baseline and post-treatment, (2) between post-treatment and 3-month follow-up, and (3) between 3- and 6-month follow-up, using one-way ANOVAs. The first gain-score analysis of BDI-21 scores showed a significant group difference between the symptom severity groups [*F*(2,47) = 5.51, *p* = 0.007]. Pairwise comparisons showed that BDI-21 scores decreased more in the moderate symptom severity group during the intervention (*n* = 22, *mean gain* = –10.50, *SD* = 8.52) compared to both the no/minimal (*n* = 11, *mean gain* = –4.64, *SD* = 2.91) and mild (*n* = 17, *mean gain* = –3.23, *SD* = 7.14) symptom severity groups. In the second gain-score analysis of BDI-21 scores, a decrease or no change was observed in the mild (*n* = 17, *mean gain* = –3.88, *SD* = 5.44) and moderate (*n* = 21, *mean gain* = 0.67, *SD* = 5.69) symptom severity groups, compared with the no/minimal symptom severity group (*n* = 11, *mean gain* = 8.36, *SD* = 15.89), these differences being statistically significant [*F*(2,45) = 6.39, *p* = 0.004]. We found no symptom severity group differences in the third gain-score comparison of BDI-21 scores.

Examining the change in symptoms assessed with ADRSc, significant differences emerged in the first gain-score comparison from baseline to post-treatment [*F*(2,48) = 6.98, *p* = 0.002]. Pairwise comparisons using Dunnett’s correction showed that ADRSc scores decreased more in the severe depression group during this period (*n* = 17, *mean gain* = –11.19, *SD* = 7.58) when compared to no clinical depression (*n* = 11, *mean gain* = –4.82, *SD* = 1.67) and clinical depression groups (*n* = 23, *mean gain* = –4.61, *SD* = 5.65). No depression severity group differences were observed for the other two gain-score analyses.

### Predictors of remission from depression

To identify predictors of remission from depression, we analyzed whether the selected baseline variables predicted remission according to BDI-21 (sum score < 10; Beck et al., 1988) and ADRSc (sum score < 15; Revah-Levy et al., 2007) at post-treatment, at 3- and at 6-month follow-up, and whether differences were already apparent at baseline. At post-treatment, 58% (29/50), at 3-month follow-up 56% (27/48), and at 6-month follow-up 67% (32/48) of participants achieved remission as defined by the BDI-21 total score. The respective rates of remission as defined by the ADRSc score were at post-treatment 78% (40/51), at 3-month follow-up 76% (37/49), and 86% (42/49) at 6-month follow-up.

Examining baseline level of self-reported depressive symptoms associated with the predictor variables, we found that among participants with comorbid anxiety disorder, baseline BDI-21 scores were already higher than among those without comorbid anxiety disorder. Three baseline variables predicted belonging to the remission group according to BDI-21 on at least one of the examined time points. These variables were gender, comorbid anxiety disorder, and sleep difficulties (Table 3). Boys achieved remission more often than girls at post-treatment and at 3-month follow-up. The probability of remission was higher among participants with no comorbid anxiety disorder than among those with anxiety disorder at post-treatment, 3-month follow-up, and at 6-month follow-up. Adolescents without baseline sleep difficulties were more likely to achieve remission than those with sleep difficulties at post-treatment and at 3-month follow-up.

**TABLE 3:** Clinical remission according to Beck Depression Inventory (BDI)-21 (BDI-21 < 10).

		Differences in baseline BDI-21 score (*N* = 55)			Clinical remission at post-treatment (*N* = 50^a^)			Clinical remission at 3-month follow-up (*N* = 48^a^)			Clinical remission at 6-month follow-up (*N* = 48^a^)		
					
		Variables *n*	BDI-21 mean (*SD*)	*t*(*df*)	BDI-21 > 10 (*n* = 29)	BDI-21 < 10 (*n* = 21)	*t*^(^*^df^*^)^/*x*^2^	BDI-21 > 10 (*n* = 27)	BDI-21 < 10 (*n* = 21)	*t*^(^*^df^*^)^/*x*^2^	BDI-21 > 10 (*n* = 32)	BDI-21 < 10 (*n* = 16)	*t* ^(^*^df^*^)^/*x*^2^
Demographic variables	Age mean (*SD*)	14.53 (0.78) <29	16.83 (10.06)	*t*(53) = 0.55	14.35 (0.68)	14.69 (0.70)	*t*(48) = 1.69	14.44 (0.66)	14.61 (0.71)	*t*(46) = –0.86	14.46 (0.62)	14.46 (0.84)	*t*(46) = –0.03
		14.53 (0.78) >26	18.19 (8.09)										
	Gender	Female 43	18.39 (7.96)	*t*(53) = –1.43	20 (50.0%)	20 (50.0%)	*x*^2^(1) = 5.26*^b^	20 (52.6%)	18 (47.4%)	*x*^2^(1) = 5.85*^b^	23 (60.5%)	15 (39.5%)	*x*^2^(1) = 3.09^b^
		Male 12	14.17 (9.29)		9 (90.0%)	1 (10.0%)		9 (90.0%)	1 (90.0%)		9 (90.0%)	1 (10%)	
Psychosocial variables	Family constellation	Both 28	16.54 (9.39)	*t*(53) = –0.77	16 (61.5%)	10 (38.5%)	*x*^2^(1) = 0.28	9 (34.6%)	17 (65.4%)	*x*^2^(1) = 1.92	18 (69.2%)	8 (30.8%)	*x*^2^(1) = 0.17
		One 27	18.44 (8.91)		13 (54.2%)	11 (45.8%)		12 (54.5%)	10 (45.5%)		14 (63.6%)	8 (36.4%)	
	Close relationship with parents	Yes 41	16.22 (9.00)	*t*(53) = –1.78	23 (59.0%)	16 (41.0%)	*x*^2^(1) = 0.07	23 (59.0%)	16 (41.0%)	*x*^2^(1) = 0.63^b^	28 (71.8%)	11 (28.2%)	*x*^2^(1) = 2.46^b^
		No 14	21.14 (8.76)		6 (54.5%)	5 (45.5%)		4 (44.4%)	5 (55.6%)		4 (44.4%)	5 (55.6%)	
Clinical variables	Comorbid anxiety disorder	Yes 16	22.81 (10.44)	*t*(53) = –2.98*	5 (33.3%)	10 (66.7%)	*x*^2^(1) = 5.52*	5 (33.3%)	10 (66.7%)	*x*^2^(1) = 4.656*	6 (42.9%)	8 (57.1%)	*x*^2^(1) = 5.04*
		Not 29	15.28 (7.64)		24 (68.6%)	11 (31.4%)		22 (66.7%)	11 (33.3%)		26 (76.5%)	8 (23.5%)	
	Sleep difficulties	Yes 10	21.80 (7.77)	*t*(53) = 1.69	2 (22.2%)	7 (77.8%)	*x*^2^(1) = 5.77*^b^	7 (77.8%)	2 (22.2%)	*x*^2^(1) = 5.21*^b^	4 (50.0%)	4 (50.0%)	*x*^2^(1) = 1.20^b^
		Not 45	16.51 (9.20)		27 (65.9%)	14 (34.1%)		25 (64.1%)	14 (35.9%)		28 (70.0%)	12 (30.0%)	

BDI-21, Beck Depression Inventory. ^a^Data missing in one case. ^b^Fisher’s exact test. *p < 0.05.

Examining baseline level of depressive symptoms as defined by ADRSc, as associated with each of the predictor variables, we observed that boys’ ADRSc scores were lower than those of girls. In addition, among participants with comorbid anxiety disorder, baseline ADRSc scores were already higher than scores among those without comorbid anxiety disorder. One baseline variable predicted belonging to remission group according to ADRSc at least on one of the examined time points. Not having sleep difficulties predicted remission as defined by ADRSc score at post-treatment. No other statistically significant predictors were found for ADRSc (Table 4).

**TABLE 4:** Clinical remission according to Adolescent Depression Rating Scale (ADRSc) (ADRSc < 15).

		Differences in baseline ADRSc score (*N* = 54^a^)			Clinical remission at post-treatment (*N* = 51)			Clinical remission at 3-month follow-up (*N* = 49)			Clinical remission at 6-month follow-up (*N* = 49)		
					
		Variables, *n*	ADRSc mean (*SD*)	*t*(*df*)	ADRSc < 15 (*n* = 40)	ADRSc > 15 (*n* = 11)	*t^(df^*^)^/*x*^2^	ADRSc < 15 (*n* = 37)	ADRSc > 15 (*n* = 12)	*t*^(^*^df^*^)^/*x*^2^	ADRSc < 15 (*n* = 42)	ADRSc > 15 (*n* = 7)	*t ^(df^*^)^/*x*^2^
Demographic variables	Age mean (*SD*)	14.53 <26	15.25 (6.69)	*t*(52) = 1.04	14.53 (0.74)	14.49 (0.76)	*t*(49) = 0.15	14.48 (0.69.8)	14.48 (0.78)	*t*(47) = 0.016	14.46 (0.68)	14.56 (0.90)	*t*(47) = –0.319
		14.53 >28	17.46 (8.80)										
	Gender	Female 42	17.14 (8.33)	*t*(52) = –1.48*	29 (72.5%)	11 (27.5%)	*x*^2^(1) = 3.86 ^b^	27 (69.2%)	12 (30.8%)	*x*^2^(1) = 4.075^b^	33 (84.6%)	6 (15.4%)	*x*^2^(1) = 0.188^b^
		Male 12	13.42 (4.58)		11 (100.0%)	0 (0.0%)		10 (100.0%)	0 (0.0%)		9 (90.0%)	1 (10.0%)	
Psychosocial variables	Family constellation	Both 28	14.86 (6.42)	*t*(52) = –1.44	22 (81.5%)	5 (18.5%)	*x*^2^(1) = 0.32	19 (73.1%)	7 (26.9%)	*x*^2^(1) = 0.177	23 (88.5%)	3 (11.5%)	*x*^2^(1) = 0.341^b^
		One 26	17.88 (8.88)		18 (75%)	6 (25%)		18 (78.3%)	5 (21.7%)		19 (82.6%)	4 (17.4%)	
	Close relationship with parents	Yes 48	15.44 (6.83)	*t*(52) = 1.22	32 (80%)	8 (20%)	*x*^2^(1) = 0.27^b^	30 (76.9%)	9 (23.1%)	*x*^2^(1) = 0.206^b^	35 (89.7%)	4 (10.3%)	*x*^2^(1) = 2.534^b^
		No 6	19.08 (10.04)		8 (72.7%)	3 (27.3%)		7 (70.0%)	3 (30.0%)		7 (70.0%)	3 (30.0%)	
Clinical variables	Comorbid anxiety disorders	Yes 16	19.62 (8.80)	*t*(52) = –2.09*	10 (66.7%)	5 (33.3%)	*x*^2^(1) = 1.739	9 (60.0%)	6 (40.0%)	*x*^2^(1) = 2.812	11 (73.3%)	4 (26.7%)	*x*^2^(1) = 2.706^b^
		No 28	14.92 (6.97)		30 (83.3%)	6 (16.7%)		28 (82.4%)	6 (17.6%)		31 (91.2%)	3 (8.8%)	
	Sleep difficulties	Yes 10	20.3 (9.43)	*t*(52) = 1.83	4 (40%)	6 (60%)	*x*^2^(1) = 10.87**^b^	5 (55.6%)	4 (44.4%)	*x*^2^(1) = 2.374^b^	6 (66.7%)	3 (33.3%)	*x*^2^(1) = 3.267^b^
		No 44	15.41 (7.18)		36 (87.8%)	5 (12.2%)		32 (80.0%)	8 (20.0%)		36 (90.0%)	4 (10.0%)	

ADRSc, Adolescent Depression Rating Scale, clinician version. ^a^Data missing in one case. ^b^Fisher’s exact test. *p < 0.05, **p < 0.01.

## Discussion

The main findings of the study were that younger age, male gender, close relationship with parents, mild depressive symptoms, not having comorbid anxiety disorder and not having sleep difficulties were predictors of decrease in depressive symptoms. However, none of the variables other than self-assessed depression predicted change of depression score between post-treatment and 3-month follow-up. Male gender, not having comorbid anxiety disorder, and not having sleep difficulties were predictors of remission from depression. Although younger age did not predict remission from depression as the analyzing method was not able to differentiate individual changes enough, it expectedly predicted larger decrease in depressive symptoms according to self-assessed depression during intervention, not in follow-ups This finding is congruent with several previous studies (Jayson et al., 1998; Curry et al., 2006; Emslie et al., 2011; Abbott et al., 2019). Contrasting our finding, Mufson et al. (2004) reported older age predicting a better outcome after IPT-A for depression, and Weersing et al. (2006) found that age had no effect on outcome in a brief trial of behavior therapy for pediatric anxiety and depression in the primary care. While age may not be a predictor of outcome across different types of psychotherapy for adolescent depression, as suggested by Weisz et al. (2006), its predictive role in treating different problems (e.g., disruptive behaviors), types of psychotherapy (e.g., family therapy, IPT-A, or CBT), or in interventions of varying length seems worth studying.

Our finding that male gender was a predictor of both remission and of larger decrease in depressive symptoms was unexpected. Previous research suggests that female, rather than male, gender might be a predictor of better outcome (Weisz et al., 1995; Bolton et al., 2007); or that gender has no effect on outcome (Clarke et al., 1992; Curry et al., 2006; Weisz et al., 2006; Courtney et al., 2022) of treatment for adolescent depression. Our finding may, however, also be related to females having higher depressive symptom scores at baseline. Due to small proportion of males (22%) in the present study, the finding needs to be regarded as preliminary.

Consistent with Emslie et al. (2011), reporting lower levels of family stress or conflict, and higher family involvement to predict better treatment outcomes in adolescent depression we found that close relationship with parents at baseline predicted good outcome as it was associated with a larger decrease in depressive symptoms. The finding is also consistent with studies reporting different aspects of impaired family functioning, or high levels of family conflict (Asarnow et al., 2009; Feeny et al., 2009; Gunlicks-Stoessel et al., 2010) predicting poorer treatment outcome or longer time to recovery (Rohde et al., 2006) in the treatment of adolescent depression. Interestingly, in our study the positive effect of close relationship with parents became evident during the 3–6-month follow-up, suggesting that a close relationship may support recovery after the intervention. In the meta-analysis by Sun et al. (2019), parental involvement in youth psychotherapy was found to be a predictor of positive effects at follow-up. Taken together, these findings support the view that including a family intervention component in youth depression treatments might improve treatment outcomes (Oud et al., 2019).

The finding that more severe depressive symptoms at baseline predicted a larger decrease in depressive symptoms during treatment and follow-ups is probably at least partly due to the larger possibility for improvement for those with higher baseline symptom levels. Depressive symptoms also decreased more among adolescents classified as suffering from more severe depression in comparison to those with milder depression, but they ended up having higher depressive symptom scores and were less likely to reach remission. Thus, as expected, and in line with several previous studies (Brent et al., 1998; Jayson et al., 1998; Asarnow et al., 2009; Wilkinson et al., 2009), adolescents with less severe depression at baseline were more likely to reach remission.

Our finding may partly be accounted for by the definition of remission, as participants with more severe baseline depressive symptoms had to achieve a larger decrease in symptoms to reach a subclinical level. Further, it may be, that adolescents with more severe depression are not able to benefit from therapy early in treatment due to dysfunction in cognition, before some symptomatic improvement has taken place (Emslie et al., 2011). As Kunas et al. (2021) suggest, symptom severity may also be associated with other clinical characteristics that interfere with successful therapy, such as higher trait anxiety or low levels of self-efficacy.

In concordance with previous studies (Brent et al., 1998; Curry et al., 2006; Young et al., 2006; Wilkinson et al., 2009; Abbott et al., 2019), and supporting our hypothesis, comorbid anxiety disorders predicted poorer treatment outcome as they were associated with smaller decreases in clinician rated depressive symptoms between baseline to post-treatment and self-assessed non-remission between all studied time points. Brent et al. (1998) found that comorbid anxiety had a moderator effect on outcome of psychotherapy; cognitive therapy which focused on restructuring cognitive distortions was more effective with depressed youth with comorbid anxiety than was supportive therapy or family therapy. In the development of psychotherapy models for adolescent depression, inclusion of cognitive and behavioral therapy techniques also targeting anxiety symptomatology, or showing transdiagnostic effect (Brent et al., 2020), may be needed.

In the present study, baseline sleep difficulties predicted both smaller decrease according to clinician rated depressive symptoms and clinician rated non-remission between baseline and post-treatment and self-assessed non-remission up to 3-month follow-up. This finding is in line with results of two medication trials reporting higher risk of non-response and non-remission after acute treatment in adolescents with sleep dysfunction (Emslie et al., 2012), and those indicating a higher relapse risk (Kennard et al., 2018) for adolescents with sleep difficulties who initially showed response to fluoxetine treatment.

Of psychotherapy studies, in line with our findings, the IPT-A vs. treatment as usual study by McGlinchey et al. (2017) reported sleep disturbance being associated with more severe depressive symptoms and interpersonal stress at post-treatment in both treatment arms. McGlinchey et al. (2017) suggest that sleep disturbance may signal a more severe form of depression in teens. Our finding that adolescents with baseline sleep difficulties had more severe baseline depressive symptoms supports their view.

It seems likely that a brief, six-session depression intervention may not be effective enough for adolescents with both depression and sleep difficulties, and additional treatment modules on sleep disturbance and its underlying mechanisms are needed. This kind of therapy adaptation is supported by results from a pilot study by Clarke et al. (2015) among adolescents with depression and insomnia, showing that combining CBT for depression with CBT for insomnia resulted in larger treatment effects than CBT alone for improving both sleep and depression.

### Strengths and limitations

The study was conducted in adolescents’ natural environment, treatments being implemented in the school health and welfare services and provided by professionals working in these services. Studying treatments in natural community environments increases the ecological validity of the results (Rich et al., 2014). Another strength of the present study is the use of standardized and validated assessment instruments. The K-SADS-5, a widely used diagnostic interview in adolescents with well-established reliability and validity (Lauth et al., 2010), was used both at baseline and at follow-ups. Both BDI-21 and ADRSc have good psychometric properties in adolescents.

Several limitations need to be considered. First, due to the small study sample the possibility of type II error cannot be ruled out, and we were not able to explore treatment moderators or analyze all possible predictors and assessment timepoints in one model. Due to sample size, model fit for the initial LCS models were unsatisfactory as multiple variables were included to explain the change in depression scores. However, when only the statistically significant variables were included, a good model fit was achieved. Second, as only the effect of baseline depressive symptom severity was controlled for in the analyses, the effect of other possible confounders cannot be excluded. Third, since almost 80% of the sample was female, our findings considering males should be interpreted with caution. This is notable especially in crosstabulation, when the number of cases in one cell was low, also some cells in sleep difficulties included fewer than five adolescents. Yet, both gender and sleep difficulties remained strong predictors throughout the analyses. However, many variable was a predictor only in part of the analyses and studied time points. Fourth, it may be that the adolescents identified as having sleep difficulties, defined as experiencing problems nearly every night in this study, are at the more severe end of sleep difficulties.

### Clinical implications

Baseline symptom severity according to all analyses, anxiety disorder comorbidity and sleep difficulties according to clinician-rated depression symptoms, predicted poorer treatment response after a brief intervention for adolescent depression. Therefore, professionals working in primary care settings should consider more intensive or longer interventions for adolescents with high severity of depressive symptoms, comorbid anxiety disorders, or sleep difficulties. For these adolescents, modified, more intensive and longer psychological treatments, or consideration of adding psychopharmacological treatment may be needed. Our findings also highlight the importance of a thorough baseline assessment. Brief, targeted interventions in community settings hold promise for decreasing depression symptoms for adolescents with mild and moderate, non-complicated depressive disorders.

## Data availability statement

The data analyzed in this study is subject to the following licenses/restrictions: The datasets presented in this article are not readily available because the data can be used only in the studies in question by the approval from the Ethics Committee of the Helsinki and Uusimaa Hospital District and the permission from participants to use the data. Requests to access these datasets should be directed to corresponding author.

## Ethics statement

The studies involving human participants were reviewed and approved by Ethics Committee of the Helsinki and Uusimaa Hospital District. Written informed consent to participate in this study was provided by the participants’ legal guardian/next of kin.

## Author contributions

PP, MM, KR, and VG contributed to conception and design of the study. PP performed the statistical analysis and interpreted the results under supervision of MT. PP wrote the first draft of the manuscript. MM and KR provided their expertise by revising further. MM, KR, and VG critically modified the manuscript drafts. All authors contributed to manuscript revision and approved the submitted version.
