# Supplementary material for: Predictors of outcome after a time-limited psychosocial intervention for adolescent depression
Source: Front Psychol. 2022 Nov 2;13:955261. doi: 10.3389/fpsyg.2022.955261 (PMC9667940; doi:10.3389/fpsyg.2022.955261)
Supplement: Supplementary file 1 [file Table_1.docx]

Supplementary Table 1. The standardized estimates of initial LCS models for BDI-21 and ADRSc

| Models | Post BDI-21  *β* (*s.e*.)  R2 | 3-mo BDI-21  *β* (*s.e*.)  R2 | 6-mo BDI-21  *β (s.e*.)  R2 | Post ADRSc  *β* (*s.e*.)  R2 | 3-mo ADRSc  *β* (*s.e*.)  R2 | 6-mo ADRSc  *β* (*s.e*.)  R2 |
| --- | --- | --- | --- | --- | --- | --- |
| Depression score | -.48(.08)***  .23 | -.39(.12)**  .1521 | -.68(.08)***  .4624 | -.57(.09)***  .3249 | -.28(.21)  .0784 | -.76(.08)***  .5776 |
| Intervention group | .07(.12)  .005 | -.01(.12)  .0001 | .05(.09)  .0025 | .17(.11)  .0289 | -.17(.13)  .0289 | -.02(.10)  .0004 |
| Age | .29(.13)*  .08 | .10(.11)  .01 | -.04(.12)  .0016 | .10(.12)  .01 | -.01(.13)  .0001 | -.00(.09)  0 |
| Gender | .37(.12)**  .14 | .13(.08)  .0169 | .06(.07)  .0036 | .28(.10)**  .0784 | .21(.11)  .0441 | -.02(.10)  .0004 |
| Family constellation | .05(.12)  .0025 | -.07(.16)  .0049 | .09(.10)  .0081 | .06(.13)  .0036 | .06(.18)  .0036 | .02(.10)  .0004 |
| Close relationship with parents | .06(.10)  .0036 | .08(.18)  .0064 | -.28(.10)**  .0784 | -.04(.12)  .0016 | .04(.19)  .0016 | -.19(.10)  .0361 |
| Comorbid  anxiety disorders | .02(.16)  .0004 | -.09(.15)  .0081 | -.04(.15)  .0016 | -.23(12)  .0529 | .18(.22)  .0324 | .01(.12)  .0001 |
| Sleep difficulties | .08(.17)  .006 | .27(.21)  .0729 | .21(.13)  .0441 | .36(.13)**  .1296 | -.29(.23)  .0841 | .14(.13)  .0196 |
|  |  |  |  |  |  |  |
| R2 | .40 | .25 | .57 | .50 | .19 | .63 |
|  |  |  |  |  |  |  |
| Model fit | ꭓ^2^(7)=13.96, *p* > .05, *RMSEA*=.13, *CFI* = .77, *SRMR* = .16 | ꭓ^2^(7)=18.15, *p* < .05, *RMSEA*=.18, *CFI* = .22, *SRMR* = .18 | ꭓ^2^(7)=11.54, *p* > .05, *RMSEA*=.12, *CFI*=.79, *SRMR* = .17 | ꭓ^2^(7)=11.70,  *p* > .05, *RMSEA* =.11, *CFI* = .88, *SRMR* = .15 | ꭓ^2^(7)=19.90,  *p* < .05,  *RMSEA*=.19, *CFI* = .08, *SRMR* = .18 | (ꭓ^2^(7)=6.51, *p* < .05,  *RMSEA*=.00, *CFI* = 1.00, *SRMR* = .11 |

*Post = change in depression score from baseline to post-treatment, 3-mo = change in depression score from post-treatment to 3-month follow-up, 6-mo =*

*change in depression score from 3-month follow-up to 6-month follow-up*

*BDI-21* Beck Depression Inventory, *ADRSc* Adolescent Depression Rating Scale clinician version,

*β= S*tandardized estimate for regression*, s.e*.= standard error, R2 = amount of explained variance

**p* < .05, ***p* < .01, ****p* < .001
